# Association of Frequent Aspirin Use With Ovarian Cancer Risk According to Genetic Susceptibility

**DOI:** 10.1001/jamanetworkopen.2023.0666

**Published:** 2023-02-24

**Authors:** Lauren M. Hurwitz, Penelope M. Webb, Susan J. Jordan, Jennifer A. Doherty, Holly R. Harris, Marc T. Goodman, Yurii B. Shvetsov, Francesmary Modugno, Kirsten B. Moysich, Joellen M. Schildkraut, Andrew Berchuck, Hoda Anton-Culver, Argyrios Ziogas, Usha Menon, Susan J. Ramus, Anna H. Wu, Celeste Leigh Pearce, Nicolas Wentzensen, Shelley S. Tworoger, Paul D. P. Pharoah, Britton Trabert

**Affiliations:** 1 Division of Cancer Epidemiology and Genetics, National Cancer Institute, Rockville, Maryland; 2 Population Health Program, QIMR Berghofer Medical Research Institute, Brisbane, Queensland, Australia; 3 School of Public Health, University of Queensland, Brisbane, Queensland, Australia; 4 Department of Population Health Sciences, Huntsman Cancer Institute, University of Utah, Salt Lake City; 5 Program in Epidemiology, Division of Public Health Sciences, Fred Hutchinson Cancer Research Center, Seattle, Washington; 6 Department of Epidemiology, School of Public Health, University of Washington, Seattle; 7 Samuel Oschin Comprehensive Cancer Institute, Cedars-Sinai Medical Center, Los Angeles, California; 8 Cancer Epidemiology Program, University of Hawaii Cancer Center, Honolulu; 9 Division of Gynecologic Oncology, Department of Obstetrics, Gynecology, and Reproductive Sciences, University of Pittsburgh School of Medicine, Pittsburgh, Pennsylvania; 10 Department of Epidemiology, University of Pittsburgh Graduate School of Public Health, Pittsburgh, Pennsylvania; 11 Women’s Cancer Research Program, Magee-Womens Research Institute and Hillman Cancer Center, Pittsburgh, Pennsylvania; 12 Department of Cancer Prevention and Control, Roswell Park Comprehensive Cancer Center, Buffalo, New York; 13 Department of Epidemiology, Emory University Rollins School of Public Health, Atlanta, Georgia; 14 Division of Gynecologic Oncology, Duke University Medical Center, Durham, North Carolina; 15 Department of Epidemiology, University of California, Irvine; 16 MRC Clinical Trials Unit, Institute of Clinical Trials and Methodology, University College London, London, United Kingdom; 17 School of Clinical Medicine, Faculty of Medicine and Health, University of New South Wales, Sydney, Australia; 18 Adult Cancer Program, Lowy Cancer Research Centre, University of New South Wales, Sydney, Australia; 19 Keck School of Medicine, Department of Population and Public Health Sciences, University of Southern California, Los Angeles; 20 Department of Epidemiology, University of Michigan School of Public Health, Ann Arbor; 21 Department of Cancer Epidemiology, H. Lee Moffitt Cancer Center and Research Institute, Tampa, Florida; 22 Department of Public Health and Primary Care, Centre for Cancer Genetic Epidemiology, University of Cambridge, Cambridge, United Kingdom; 23 Centre for Cancer Genetic Epidemiology, Department of Oncology, University of Cambridge, Cambridge, United Kingdom; 24 Huntsman Cancer Institute, Department of Obstetrics and Gynecology, University of Utah, Salt Lake City

## Abstract

**Importance:**

Frequent aspirin use is associated with reduced ovarian cancer risk, but it is unknown whether genetic factors modify this association. Understanding effect modifiers is important given that any use of aspirin for ovarian cancer chemoprevention will likely need to focus on specific higher-risk subgroups.

**Objective:**

To evaluate whether the association between frequent aspirin use and ovarian cancer is modified by a polygenic score (PGS) for nonmucinous ovarian cancer.

**Design, Setting, and Participants:**

We pooled individual-level data from 8 population-based case-control studies from the Ovarian Cancer Association Consortium conducted in the US, UK, and Australia between 1995 and 2009. We included case patients and control participants with both genetic data and data on frequent aspirin use. Case patients with mucinous ovarian cancer were excluded. Data were analyzed between November 1, 2021, and July 31, 2022.

**Exposures:**

Frequent aspirin use, defined as daily or almost daily use for 6 months or longer.

**Main Outcomes and Measures:**

The main outcome was nonmucinous epithelial ovarian cancer. We used logistic regression to estimate odds ratios (ORs) and 95% CIs and likelihood ratio tests to investigate effect modification by the PGS.

**Results:**

There were 4476 case patients with nonmucinous ovarian cancer and 6659 control participants included in this analysis. At study enrollment, the median (IQR) age was 58 (50-66) years for case patients and 57 (49-65) years for control participants. Case patients and control participants self-reported that they were Black (122 [3%] vs 218 [3%]), White (3995 [89%] vs 5851 [88%]), or of other race and ethnicity (348 [8%] vs 580 [9%]; race and ethnicity were unknown for 11 [0%] vs 10 [0%]). There were 575 case patients (13%) and 1030 control participants (15%) who reported frequent aspirin use. The 13% reduction in ovarian cancer risk associated with frequent aspirin use (OR, 0.87 [95% CI, 0.76-0.99]) was not modified by the PGS. Consistent ORs were observed among individuals with a PGS less than (0.85 [0.70-1.02]) and greater than (0.86 [0.74-1.01]) the median. Results were similar by histotype.

**Conclusions and Relevance:**

The findings of this study suggest that genetic susceptibility to ovarian cancer based on currently identified common genetic variants does not appear to modify the protective association between frequent aspirin use and ovarian cancer risk. Future work should continue to explore the role of aspirin use for ovarian cancer prevention among individuals who are at higher risk for ovarian cancer.

## Key Points

**Question:**

Is the association between frequent aspirin use and reduced risk of ovarian cancer modified by genetic susceptibility to ovarian cancer, assessed using a polygenic score (PGS)?

**Findings:**

In this pooled analysis of 8 case-control studies from the Ovarian Cancer Association Consortium, including 4476 case patients and 6659 control participants, there was no evidence of effect modification by the PGS. Consistent associations between frequent aspirin use and reduced risk of ovarian cancer were observed for individuals with a PGS less than and greater than the median.

**Meaning:**

The findings of this study suggest that frequent aspirin use may lower risk of ovarian cancer regardless of an individual’s genetic susceptibility to ovarian cancer.

## Teaser

This case-control study pools and analyzes individual-level data from 8 Ovarian Cancer Association Consortium case-control studies to evaluate whether a polygenic score modifies the association of frequent aspirin use with ovarian cancer.

## Introduction

Ovarian cancer is a highly fatal gynecologic malignant neoplasm with few known modifiable risk factors.^1^ Evidence suggests that aspirin may protect against the development of ovarian cancer, particularly when used frequently (daily or near daily).^2,3^ In a pooled analysis of 17 cohort and case-control studies, frequent aspirin use was associated with a 13% reduced risk of ovarian cancer, with no significant heterogeneity by study design or ovarian cancer histotype.^4^

While aspirin is a promising chemopreventive agent for ovarian cancer, its use remains limited by several factors. First, serious adverse events can occur with aspirin use, including gastric ulcer and hemorrhagic stroke^5^; although rare, these risks are nonnegligible. Second, the incidence of ovarian cancer in the general population is low; thus, the number needed to treat to prevent 1 case of ovarian cancer is high.^4^ Targeting chemoprevention programs to individuals at higher risk of ovarian cancer could reduce the number needed to treat and improve the benefit-harm profile.^6^

We previously investigated whether individuals at increased risk of ovarian cancer due to epidemiologic risk factors (endometriosis, obesity, family history of breast or ovarian cancer, nulliparity, no oral contraceptive use, no tubal ligation) might benefit from frequent aspirin use. We did not observe effect modification by these individual risk factors or an epidemiologic risk factor score calculated as the number of epidemiologic risk factors.^4^ In the current analysis, we expanded our evaluation to test whether the association of frequent aspirin use with ovarian cancer is modified by genetic susceptibility to ovarian cancer, assessed using a polygenic score (PGS) based on common genetic variants.^7^

## Methods

### Study Design and Population

For this case-control study, we pooled data from the following 8 population-based case-control studies from the Ovarian Cancer Association Consortium (OCAC): the Australian Ovarian Cancer Study,^8^ the Diseases of the Ovary and Their Evaluation Study,^9,10^ the Hawaii Ovarian Cancer Study,^11,12^ the Hormones and Ovarian Cancer Prediction Study,^13^ the North Carolina Ovarian Cancer Study,^14,15^ the University of California, Irvine Ovarian Cancer Study,^16^ the UK Ovarian Cancer Population Study,^17^ and the University of Southern California Study of Lifestyle and Women’s Health^18^ (eTable 1 in Supplement 1). Participants were enrolled between 1995 and 2009; eligibility criteria and methods of case and control ascertainment for each study have been previously described.^8,9,10,11,12,13,14,15,16,17,18^ These 8 OCAC studies were included because they collected data on self-reported frequency of aspirin use, as described in eTable 1 in Supplement 1. For this analysis, frequent aspirin use (yes or no) was harmonized across the studies to indicate daily or almost daily use for 6 months or longer, to the extent possible. We focused specifically on frequent aspirin use, as this was the pattern of aspirin use most consistently associated with reduced ovarian cancer risk in prior analyses.^2,3^ Other covariates were harmonized as previously described.^2^ All participants provided either written informed consent or implicit consent through return of the study questionnaire. Participating studies obtained institutional review board (IRB) approval at their respective institutions, and the OCAC Coordinating Center (Duke University) received IRB approval from its institution and participating registries as required for data acquisition, pooling, and harmonization. This study followed the Strengthening the Reporting of Observational Studies in Epidemiology (STROBE) reporting guideline.

Within these 8 studies, 86% of case patients and control participants had genotype data available. Sample collection, genotyping, and quality control were conducted as described previously.^19^ Genetic susceptibility to ovarian cancer was summarized using a PGS previously developed within 63 OCAC studies and validated in external populations.^7^ We used the PGS developed using the stepwise method (22 single-nucleotide variants; eTable 2 in Supplement 1). Because this PGS was developed for nonmucinous epithelial ovarian cancer, we only included case patients with nonmucinous cancer in our analysis (242 case patients were excluded).

### Statistical Analysis

We used logistic regression to estimate odds ratios (ORs) and 95% CIs for the associations between frequent aspirin use and nonmucinous ovarian cancer. Associations were estimated overall and by quantiles of the PGS based on the PGS distribution in the controls. Given the low prevalence of ovarian cancer, ORs were assumed to estimate the relative risk. The likelihood ratio test was used to test for statistical interaction. Polytomous logistic regression, with controls as the reference group, was used to estimate associations by ovarian cancer histotype. Models were adjusted for age (continuous), study site, interaction of age and site, self-reported race and ethnicity (Black, White, other, or unknown), parity (parous, nulliparous, or unknown), duration of oral contraceptive use (none, <5 years, 5-9 years, ≥10 years, or unknown), menopausal status (premenopausal, postmenopausal, unknown), and obesity (yes [body mass index ≥30 kg/m^2^], no, or unknown). Missing covariate information was minimal (<3% for most covariates; Table 1). Analyses were conducted in Stata, version 17 (StataCorp LLC). All tests were 2 sided, and *P* < .05 was considered statistically significant. Statistical analyses were performed between November 1, 2021, and July 31, 2022.

**Table 1.** Characteristics of Case Patients With Nonmucinous Ovarian Cancer and Control Participants From 8 Studies From the OCAC

Characteristic	No. (%)	
	Case patients (n = 4476)	Control participants (n = 6659)
Age, y		
<50	942 (21)	1663 (25)
50-59	1445 (32)	2119 (32)
60-69	1338 (30)	1934 (29)
≥70	701 (16)	943 (14)
OCAC study		
Australian Ovarian Cancer Study	1004 (22)	1252 (19)
Diseases of the Ovary and Their Evaluation Study	993 (22)	1623 (24)
Hawaii Ovarian Cancer Study	211 (5)	466 (7)
Hormones and Ovarian Cancer Prediction Study	557 (12)	1250 (19)
North Carolina Ovarian Cancer Study	678 (15)	829 (12)
University of California, Irvine Ovarian Cancer Study	279 (6)	179 (3)
UK Ovarian Cancer Population Study	454 (10)	574 (9)
University of Southern California Study of Lifestyle and Women’s Health	300 (7)	486 (7)
Histotype		
High-grade serous	2584 (58)	NA
Low-grade serous	140 (3)	NA
Endometrioid	688 (15)	NA
Clear cell	375 (8)	NA
Other	680 (15)	NA
Race and ethnicity		
Black	122 (3)	218 (3)
White	3995 (89)	5851 (88)
Other^a^	348 (8)	580 (9)
Not reported	11 (0)	10 (0)
Parity		
Parous	3443 (77)	5701 (86)
Nulliparous	947 (21)	912 (14)
Not reported	86 (2)	46 (1)
Frequent aspirin use		
No	3901 (87)	5629 (85)
Yes	575 (13)	1030 (15)
Duration of oral contraceptive use, y		
Never	1629 (36)	1729 (26)
<5	1524 (34)	2315 (35)
5-<10	634 (14)	1224 (18)
≥10	539 (12)	1288 (19)
Not reported	150 (3)	103 (2)
Menopausal status		
Postmenopause	3241 (72)	4544 (68)
Premenopause	1083 (24)	1943 (29)
Not reported	152 (3)	172 (3)
Obesity		
No	2848 (64)	4503 (68)
Yes^b^	1054 (24)	1542 (23)
Not reported	574 (13)	614 (9)

Abbreviations: NA, not applicable; OCAC, Ovarian Cancer Association Consortium.

^a^ Could include self-identified Asian (asked as a general category or by category, including Chinese, Filipino, Hawaiian, Japanese, Korean, other Asian, other Pacific Islander), multiple races and ethnicities, or other race.

^b^ Defined as body mass index ≥30 kg/m^2^.

## Results

This study included 4476 case patients and 6659 control participants. At study enrollment, the median (IQR) age was 58 (50-66) years for case patients and 57 (49-65) years for control participants. Case patients and control participants self-reported that they were Black (122 [3%] vs 218 [3%]), White (3995 [89%] vs 5851 [88%]), or of other race and ethnicity (348 [8%] vs 580 [9%]; race and ethnicity were unknown for 11 [0%] vs 10 [0%]). Among the case patients, histotypes were as follows: high-grade serous (2584 [58%]), low-grade serous (140 [3%]), endometrioid (688 [15%]), clear cell (375 [8%]), and other or unknown epithelial (680 [15%]) cancer (Table 1). Case patients and control participants also primarily reported being parous and postmenopausal (Table 1). A total of 575 case patients (13%) and 1030 control participants (15%) reported frequent aspirin use.

Consistent with previous analyses that included mucinous cases, frequent aspirin use was associated with a 13% reduced risk of nonmucinous ovarian cancer (OR, 0.87 [95% CI, 0.76-0.99]) (Figure). The associations did not differ by PGS categories (all *P* interactions >.05) (Figure and eTable 3 in Supplement 1). Similar associations between frequent aspirin use and ovarian cancer were observed for individuals with a PGS less than (OR, 0.85 [95% CI, 0.70-1.02]) and greater than (0.86 [0.74-1.01]) the median, although no association was observed for individuals in the highest quintile of the PGS (1.02 [0.80-1.30]; Figure). Risk reductions were greatest for high-grade serous and endometrioid tumors (Table 2), and there was no evidence of effect modification by the PGS in histotype-specific analyses (all *P* interactions >.05) (Table 2) or by the joint classification of the PGS and epidemiologic risk factor score (*P* interaction = .64) (eTable 4 in Supplement 1).

**Figure.**
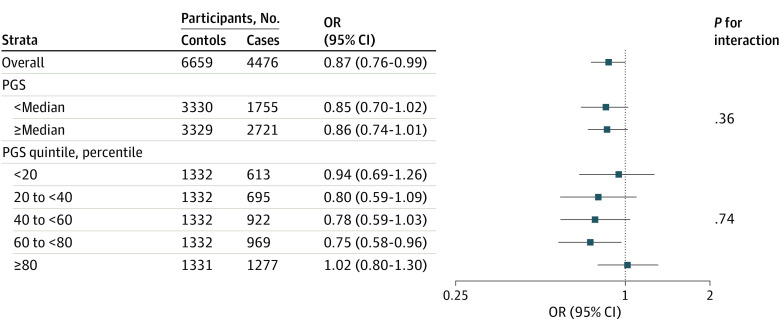
Associations Between Frequent Aspirin Use and Nonmucinous Epithelial Ovarian Cancer Risk Within Strata of Polygenic Score (PGS) Odds ratios (ORs) and 95% CIs were calculated from a logistic regression model adjusted for age, site, interaction between age and site, race and ethnicity, parity, duration of oral contraceptive use, menopausal status, and obesity (body mass index ≥30 kg/m^2^). The OR for a 1-SD increase in PGS equals 1.32 (95% CI, 1.26-1.37). The *P* interaction between frequent aspirin use and PGS treated continuously is .43.

**Table 2.** Associations Between Frequent Aspirin Use and Nonmucinous Epithelial Ovarian Cancer Risk by Histotype Within Strata of Polygenic Score^a^

Strata	No. of control participants	High-grade serous		Endometrioid			Clear cell			Other epithelial	
		No. of case patients	OR (95% CI)^b^	No. of case patients	OR (95% CI)^b^	No. of case patients		OR (95% CI)^b^	No. of case patients		OR (95% CI)^b^
Overall	6659	2584	0.83 (0.72-0.95)	688	0.73 (0.56-0.96)	375		1.00 (0.72-1.38)	680		0.98 (0.77-1.23)
PGS median											
Less than median	3330	923	0.83 (0.66-1.04)	305	0.72 (0.48-1.08)	190		0.83 (0.50-1.36)	270		1.00 (0.69-1.45)
Equal to or greater than median	3329	1661	0.84 (0.70-1.01)	383	0.76 (0.53-1.08)	185		1.22 (0.79-1.90)	410		0.97 (0.72-1.32)
*P* value for interaction^c^	NA	NA	.79	NA	.54	NA		.31	NA		.87

Abbreviations: NA, not applicable; OR, odds ratio; PGS, polygenic score.

^a^ Low-grade serous ovarian cancers were excluded due to the low number of case patients.

^b^ Adjusted for age, site, interaction between age and site, race and ethnicity, parity, duration of oral contraceptive use, menopausal status, and obesity.

^c^ Interaction between frequent aspirin use and the PGS treated continuously. *P* heterogeneity by histotype equals 0.31 for individuals with a PGS less than the median and 0.26 for individuals with a PGS equal to or greater than the median.

## Discussion

In this pooled analysis of 8 case-control studies, we observed consistent protective associations between frequent aspirin use and nonmucinous ovarian cancer across strata of genetic susceptibility to ovarian cancer. These results suggest that inherited genetic susceptibility to ovarian cancer based on currently identified common genetic variants does not modify the protective association between frequent aspirin use and ovarian cancer. The only stratum with no protective association was individuals with a PGS greater than the 80th percentile, but the CI for the association in this stratum did not preclude a 13% risk reduction; given the overall lack of evidence for effect modification, the association for this subgroup will need to be assessed in additional studies before concluding that it is null. Risk reductions were otherwise maintained in individuals with a PGS greater than the median, including for high-grade serous and endometrioid cancers, suggesting that research could further evaluate subgroups of higher-risk individuals to improve the risk-benefit profile of aspirin for chemoprevention. Although we did not observe effect modification on the multiplicative scale, future prospective studies are needed to estimate the absolute benefit of frequent aspirin use for individuals at higher risk of ovarian cancer and to weigh the benefits and harms for all conditions affected by aspirin.

### Limitations

This study has some limitations. The case-control design was retrospective and potentially limited by confounding and recall bias. However, we carefully adjusted for known potential confounders, and case-control and prospective cohort risk estimates of the association of aspirin with ovarian cancer were similar in our previous study,^4^ suggesting minimal recall bias. We only included the subset of participants with genetic data available, but the association of aspirin with ovarian cancer was nearly identical in this subset and the full case-control population,^4^ suggesting no systematic differences. We were unable to test for effect modification by pathogenic variants (ie, *BRCA1*/*BRCA2*); randomized clinical trials of aspirin use in these specific subgroups are ongoing.^20^ This study leveraged harmonized genetic and epidemiologic data from 8 ovarian cancer studies, a data resource that allowed for assessment of the association of aspirin with ovarian cancer across strata of the PGS.

## Conclusions

The findings of this case-control study suggest that frequent aspirin use reduces the risk of nonmucinous ovarian cancer—including high-grade serous and endometrioid ovarian cancer—across most strata of genetic risk based on a PGS, including among individuals with a PGS greater than the median. This work expands on the evidence base to suggest that chemoprevention programs could target individuals at higher risk of ovarian cancer, as defined by epidemiologic risk factors, polygenic risk, or both, to improve the benefit-harm profile of frequent aspirin use for ovarian cancer prevention.

## Notes

Supplement 1. **eTable 1.** Characteristics of the 8 Case-Control Studies Included From the Ovarian Cancer Association Consortium **eTable 2.** Single-Nucleotide Variants Included in the Stepwise Polygenic Score
eReference
**eTable 3.** Associations Between Frequent Aspirin Use and Nonmucinous Epithelial Ovarian Cancer Risk Within Decile of Polygenic Score **eTable 4.** Associations Between Frequent Aspirin Use and Nonmucinous Epithelial Ovarian Cancer Risk Within Joint Strata of Polygenic Score and Ovarian Cancer Epidemiologic Risk Factor Score

Supplement 2.
Data Sharing Statement

## References

[1] American Cancer Society. Cancer Facts & Figures 2022. American Cancer Society; 2022.

[2] Trabert B, Ness RB, Lo-Ciganic WH, ; Australian Ovarian Cancer Study Group, Australian Cancer Study (Ovarian Cancer); Ovarian Cancer Association Consortium. Aspirin, nonaspirin nonsteroidal anti-inflammatory drug, and acetaminophen use and risk of invasive epithelial ovarian cancer: a pooled analysis in the Ovarian Cancer Association Consortium. J Natl Cancer Inst. 2014;106(2):djt431. doi:10.1093/jnci/djt431 24503200 PMC3924755

[3] Trabert B, Poole EM, White E, ; Ovarian Cancer Cohort Consortium (OC3). Analgesic use and ovarian cancer risk: an analysis in the Ovarian Cancer Cohort Consortium. J Natl Cancer Inst. 2019;111(2):137-145. doi:10.1093/jnci/djy100 29860330 PMC6376910

[4] Hurwitz LM, Townsend MK, Jordan SJ, . Modification of the association between frequent aspirin use and ovarian cancer risk: a meta-analysis using individual-level data from two ovarian cancer consortia. J Clin Oncol. 2022;40(36):4207-4217. doi:10.1200/JCO.21.01900 35867953 PMC9916035

[5] Whitlock EP, Burda BU, Williams SB, Guirguis-Blake JM, Evans CV. Bleeding risks with aspirin use for primary prevention in adults: a systematic review for the U.S. Preventive Services Task Force. Ann Intern Med. 2016;164(12):826-835. doi:10.7326/M15-2112 27064261

[6] Drew DA, Cao Y, Chan AT. Aspirin and colorectal cancer: the promise of precision chemoprevention. Nat Rev Cancer. 2016;16(3):173-186. doi:10.1038/nrc.2016.4 26868177 PMC6741347

[7] Dareng EO, Tyrer JP, Barnes DR, ; GEMO Study Collaborators; GC-HBOC Study Collaborators; EMBRACE Collaborators; OPAL Study Group; AOCS Group; KConFab Investigators; HEBON Investigators; OCAC Consortium; CIMBA Consortium. Polygenic risk modeling for prediction of epithelial ovarian cancer risk. Eur J Hum Genet. 2022;30(3):349-362. doi:10.1038/s41431-021-00987-7 35027648 PMC8904525

[8] Merritt MA, Green AC, Nagle CM, Webb PM; Australian Cancer Study (Ovarian Cancer); Australian Ovarian Cancer Study Group. Talcum powder, chronic pelvic inflammation and NSAIDs in relation to risk of epithelial ovarian cancer. Int J Cancer. 2008;122(1):170-176. doi:10.1002/ijc.23017 17721999

[9] Hannibal CG, Rossing MA, Wicklund KG, Cushing-Haugen KL. Analgesic drug use and risk of epithelial ovarian cancer. Am J Epidemiol. 2008;167(12):1430-1437. doi:10.1093/aje/kwn082 18390840

[10] Rossing MA, Cushing-Haugen KL, Wicklund KG, Doherty JA, Weiss NS. Menopausal hormone therapy and risk of epithelial ovarian cancer. Cancer Epidemiol Biomarkers Prev. 2007;16(12):2548-2556. doi:10.1158/1055-9965.EPI-07-0550 18086757

[11] Goodman MT, Lurie G, Thompson PJ, McDuffie KE, Carney ME. Association of two common single-nucleotide polymorphisms in the CYP19A1 locus and ovarian cancer risk. Endocr Relat Cancer. 2008;15(4):1055-1060. doi:10.1677/ERC-08-0104 18667686 PMC2663409

[12] Lurie G, Wilkens LR, Thompson PJ, . Combined oral contraceptive use and epithelial ovarian cancer risk: time-related effects. Epidemiology. 2008;19(2):237-243. doi:10.1097/EDE.0b013e31816334c5 18223481

[13] Minlikeeva AN, Freudenheim JL, Lo-Ciganic WH, . Use of common analgesics is not associated with ovarian cancer survival. Cancer Epidemiol Biomarkers Prev. 2015;24(8):1291-1294. doi:10.1158/1055-9965.EPI-15-0508 26063476 PMC4526413

[14] Moorman PG, Calingaert B, Palmieri RT, . Hormonal risk factors for ovarian cancer in premenopausal and postmenopausal women. Am J Epidemiol. 2008;167(9):1059-1069. doi:10.1093/aje/kwn006 18303003 PMC2663520

[15] Schildkraut JM, Iversen ES, Wilson MA, . Association between DNA damage response and repair genes and risk of invasive serous ovarian cancer. PLoS One. 2010;5(4):e10061. doi:10.1371/journal.pone.0010061 20386703 PMC2851649

[16] Ziogas A, Gildea M, Cohen P, . Cancer risk estimates for family members of a population-based family registry for breast and ovarian cancer. Cancer Epidemiol Biomarkers Prev. 2000;9(1):103-111. 10667470

[17] Balogun N, Gentry-Maharaj A, Wozniak EL, . Recruitment of newly diagnosed ovarian cancer patients proved challenging in a multicentre biobanking study. J Clin Epidemiol. 2011;64(5):525-530. doi:10.1016/j.jclinepi.2010.07.008 21074968

[18] Wu AH, Pearce CL, Tseng CC, Templeman C, Pike MC. Markers of inflammation and risk of ovarian cancer in Los Angeles County. Int J Cancer. 2009;124(6):1409-1415. doi:10.1002/ijc.24091 19065661 PMC4203374

[19] Phelan CM, Kuchenbaecker KB, Tyrer JP, ; AOCS study group; EMBRACE Study; GEMO Study Collaborators; HEBON Study; KConFab Investigators; OPAL Study Group. Identification of 12 new susceptibility loci for different histotypes of epithelial ovarian cancer. Nat Genet. 2017;49(5):680-691. doi:10.1038/ng.3826 28346442 PMC5612337

[20] ASA in prevention of ovarian cancer (STICs and STONEs). ClinicalTrials.gov identifier: NCT03480776. Updated December 19, 2022. Accessed April 18, 2022. https://clinicaltrials.gov/ct2/show/NCT03480776

