# Supplementary material for: Association of Frequent Aspirin Use With Ovarian Cancer Risk According to Genetic Susceptibility
Source: JAMA Netw Open. 2023 Feb 24;6(2):e230666. doi: 10.1001/jamanetworkopen.2023.0666 (PMC9958519; doi:10.1001/jamanetworkopen.2023.0666)
Supplement: Supplement 1. — eTable 1. Characteristics of the 8 Case-Control Studies Included From the Ovarian Cancer Association Consortium eTable 2. Single-Nucleotide Variants Included in the Stepwise Polygenic Score eReference eTable 3. Associations Between Frequent Aspirin Use and Nonmucinous Epithelial Ovarian Cancer Risk Within Decile of Polygenic Score eTable 4. Associations Between Frequent Aspirin Use and Nonmucinous Epithelial Ovarian Cancer Risk Within Joint Strata of Polygenic Score and Ovarian Cancer Epidemiologic Risk Factor Score [file jamanetwopen-e230666-s001.pdf]

Supplementary Online Content

Hurwitz LM, Webb PM, Jordan SJ, et al. Association of frequent aspirin use with ovarian cancer risk according to genetic susceptibility. *JAMA Netw Open*. 2023;6(2):e230666. doi:10.1001/jamanetworkopen.2023.0666

eTable 1. Characteristics of the 8 Case-Control Studies Included From the Ovarian Cancer Association Consortium

eTable 2. Single-Nucleotide Variants Included in the Stepwise Polygenic Score

eReference

eTable 3. Associations Between Frequent Aspirin Use and Nonmucinous Epithelial Ovarian Cancer Risk Within Decile of Polygenic Score

eTable 4. Associations Between Frequent Aspirin Use and Nonmucinous Epithelial Ovarian Cancer Risk Within Joint Strata of Polygenic Score and Ovarian Cancer Epidemiologic Risk Factor Score

This supplementary material has been provided by the authors to give readers additional information about their work.

© 2023 Hurwitz LM et al. *JAMA Network Open*.

eTable 1. Characteristics of the 8 Case-Control Studies Included From the Ovarian Cancer Association Consortium

Study	Location	Ascertainment period	Mean age of cases	Questionnaire item for ascertaining aspirin use	Categories for frequency of use	Prevalence of frequent aspirin use among controls
Australian Ovarian Cancer Study & Australian Cancer Study	Australia	2002-2006	59.3	How often have you taken the following over-the-counter (aspirin, paracetamol, anti-inflammatory drugs) medications during the past 5 years?	Never, occasionally, < 1/month, 1/week, 2-3/week, 4-7/week, 2+/day	6.2%
Diseases of the Ovary and their Evaluation Study	USA	2002-2009	56.2	Before reference date have you taken any of these medications (show card) 5 or more days per month for at least 6 months?	Days per month: 5–7, 8–14, >14 days but less than daily, daily, or almost daily	14.9%
Hawaii Ovarian Cancer Study	USA	2001-2008	56.9	Did you ever take an aspirin product (show card) at least 12 times a year?	Numbers of pills per day, week, or month	19.2%
Hormones and Ovarian Cancer Prediction Study	USA	2003-2008	60.2	Prior to reference date have you ever used aspirin (show card) for at least two tablets per week continuously for a period of 6 months or longer?	Numbers of pills per day, week, or month	29.8%
North Carolina Ovarian Cancer Study	USA	1999-2008	57.2	For the 5 years prior to diagnosis, did you take any of these over-the-counter medications (show card) on a regular basis for at least 3 months?	Days per month: ≤1, 2-7, 8-14, >14, daily or almost daily	9.0%
University of California, Irvine Ovarian Cancer Study	USA	1995-2005	58.0	Have you taken medication listed (aspirin, ibuprofen, acetaminophen, naproxen) regularly? By regular, we are referring to use of the drug or medication at least once a week for a year, or more than 50 pills during a one year-period.	Number of pills per week	5.6%
United Kingdom Ovarian Cancer Population Study	UK	2006-2007	60.7	Have you ever used any medication containing the drugs (aspirin, ibuprofen) on a regular basis (by regular we mean every day or almost every day for 6 months or longer)?	Every day or almost every day	15.2%
University of Southern California, Study of Lifestyle and Women's Health	USA	2000-2005	57.0	Before reference date, as an adult, did you ever take any prescription or non-prescription medicine at least 2 or more times per week for one month or longer?	Number of days per month	12.7%

© 2023 Hurwitz LM et al. *JAMA Network Open*.

eTable 2. Single-Nucleotide Variants Included in the Stepwise Polygenic Score

Single Nucleotide Polymorphisms Included in the Stepwise Polygenic Score (PGS), including their beta coefficients (weights), adapted from Supplemental Table 3 of Dareng et al. [1]

SNP	Chromosome	Position	Reference Allele	Effect Allele	Beta Coefficient	Effect Allele Frequency* (%)
1:38082122_G_A	1	38082122	G	A	0.0781813	25.4
2:177039578_G_T	2	177039578	G	T	-0.105074	68.0
3:156402487_C_T	3	156402487	C	T	0.359506	4.9
3:190531882_G_A	3	190531882	G	A	-0.0690369	30.3
5:1284135_C_T	5	1284135	C	T	0.107854	33.5
5:54476556_G_A	5	54476556	G	A	-0.0816521	73.1
8:129541931_G_A	8	129541931	G	A	-0.183233	13.0
8:128817883_A_G	8	128817883	A	G	0.0747625	45.7
8:129080657_G_C	8	129080657	G	C	-0.0613552	39.2
8:82653644_A_G	8	82653644	A	G	0.137088	6.8
9:16914716_G_A	9	16914716	G	A	-0.124223	20.5
9:16914895_A_G	9	16914895	A	G	-0.0963606	32.2
9:136155000_C_T	9	136155000	C	T	0.0973786	19.7
9:106860568_C_T	9	106860568	C	T	0.0625698	55.2
9:19098967_G_A	9	19098967	G	A	0.0650448	29.1
10:21821274_G_A	10	21821274	G	A	0.079816	33.1
17:46472432_C_G	17	46472432	C	G	0.12305	27.3
17:43670696_G_C	17	43670696	G	C	0.105612	18.6
17:41659793_G_A	17	41659793	G	A	0.439715	0.6
17:40620553_C_A	17	40620553	C	A	0.424891	0.5
19:17390291_T_C	19	17390291	T	C	0.122958	29.7
21:36080398_T_C	21	36080398	T	C	-0.0633405	40.7

*Among controls of European ancestry

eReference

[1] Dareng EO, Tyrer JP, Barnes DR, et al. Polygenic risk modeling for prediction of epithelial ovarian cancer risk. *Eur J Hum Genet.* 022;14(10):021-00987.

© 2023 Hurwitz LM et al. *JAMA Network Open*.

eTable 3. Associations Between Frequent Aspirin Use and Nonmucinous Epithelial Ovarian Cancer Risk Within Decile of Polygenic Score

	Controls	Cases	OR (95% CI)*
Decile			
1 (low)	666	290	1.15 (0.73-1.81)
2	666	323	0.77 (0.51-1.16)
3	666	342	1.02 (0.66-1.58)
4	666	353	063 (0.40-0.98)
5	666	447	0.76 (0.51-1.14)
6	666	475	0.78 (0.53-1.15)
7	666	516	0.67 (0.47-0.95)
8	666	453	0.85 (0.58-1.24)
9	666	562	0.97 (0.68-1.38)
10	665	715	1.08 (0.77-1.51)
p-int			0.82

*Adjusted for age, site, the interaction between age and site, race, parity, duration of oral contraceptive use, menopausal status, obesity

© 2023 Hurwitz LM et al. *JAMA Network Open*.

eTable 4. Associations Between Frequent Aspirin Use and Nonmucinous Epithelial Ovarian Cancer Risk Within Joint Strata of Polygenic Score and Ovarian Cancer Epidemiologic Risk Factor Score*

	Overall		PGS <median		PGS ≥median	
Risk factor score*	Controls / Cases	OR (95% CI)**	Controls / Cases	OR (95% CI)**	Controls / Cases	OR (95% CI)**
0	887/392	0.75 (0.48-1.16)	426/152	0.49 (0.23-1.08)	461/240	1.06 (0.61-1.87)
1	2,471/1,268	0.86 (0.68-1.08)	1,229/479	1.03 (0.72-1.47)	1,242/789	0.78 (0.58-1.05)
≥2	3,301/2,816	0.84 (0.73-0.98)	1,675/1,124	0.80 (0.63-1.00)	1,626/1,692	0.89 (0.73-1.09)

*Sum of the number of epidemiologic ovarian cancer risk factors (endometriosis, obesity, family history of breast or ovarian cancer, nulliparity, no oral contraceptive use, no tubal ligation)

**Adjusted for age, site, the interaction between age and site, race, parity, menopausal status, oral contraceptive use, obesity

P-interaction between frequent aspirin use and joint strata of the risk factor score and PGS = 0.64

© 2023 Hurwitz LM et al. *JAMA Network Open*.
