# Supplementary material for: Association of Frequent Aspirin Use With Ovarian Cancer Risk According to Genetic Susceptibility
Source: JAMA Netw Open. 2023 Feb 24;6(2):e230666. doi: 10.1001/jamanetworkopen.2023.0666 (PMC9958519; doi:10.1001/jamanetworkopen.2023.0666)
Supplement: Supplement 2. — Data Sharing Statement [file jamanetwopen-e230666-s002.pdf]

Data Sharing Statement

Hurwitz. Association of Frequent Aspirin Use With Ovarian Cancer Risk According to Genetic Susceptibility. *JAMA Netw Open*. Published February 24, 2023.
doi:10.1001/jamanetworkopen.2023.0666

Data
Data available: Yes
Data types: Deidentified participant data
How to access data: OncoArray germline genotype data for the Ovarian Cancer Association Consortium studies have been deposited at the European Genome-phenome Archive (EGA; https://ega-archive.org/) under accession EGAS00001002305.
When available: With publication

Supporting Documents
Document types: None

Additional Information
Who can access the data: The full individual patient data are not publicly available but can be requested through the existing data request processes of the Ovarian Cancer Association Consortium (https://ocac.ccge.medschl.cam.ac.uk/)
Types of analyses: Please contact the Ovarian Cancer Association Consortium for further information (https://ocac.ccge.medschl.cam.ac.uk/)
Mechanisms of data availability: Please contact the Ovarian Cancer Association Consortium for further information (https://ocac.ccge.medschl.cam.ac.uk/)
